# Quality of life in persons with mild cognitive impairment: a systematic review and meta-analysis

**DOI:** 10.1590/1980-5764-DN-2023-0093

**Published:** 2024-08-26

**Authors:** Priya Gopalakrishnan, Shivani Tiwari, Ravishankar Nagaraja, Gopee Krishnan

**Affiliations:** 1 Manipal Academy of Higher Education, Manipal College of Health Professions, Department of Speech and Hearing, Manipal, Karnataka, India.; 2 University of Delhi, Vallabhbhai Patel Chest Institute, Department of Biostatistics, Delhi, India.

**Keywords:** Quality of Life, Cognitive Dysfunction, Aged, Systematic Review, Meta-analysis, Qualidade de Vida, Disfunção Cognitiva, Idoso, Revisão Sistemática, Metanálise

## Abstract

The global increase in the aging population has raised concerns over various age-related conditions like dementia and mild cognitive impairment (MCI) and their consequences on the affected persons. People with MCI exhibit cognitive deficits more significantly than expected for their age and literacy level. Though the nature of this condition is considered "mild", studies have reported that even more subtle deficits can influence the quality of life (QOL).

**Objective:**

The present work aimed at exploring and comparing QOL in older adults with and without MCI through a systematic review and meta-analysis.

**Methods:**

After a detailed search of articles till May 2021 in the relevant electronic databases (PubMed Central, PubMed, Scopus, CINAHL Plus, Web of Science, ProQuest, and Cochrane) using the keywords "mild cognitive impairment", "quality of life", "old", "old aged", "aged", "older adult", "geriatrics", "healthy controls", "healthy participants", and "normal controls", we included 23 articles in the systematic review and 12 in the meta-analysis.

**Results:**

The quality of all the included articles were assessed using the Modified Downs and Black tool. Most of the studies in the systematic review demonstrated differences in QOL scores in older adults with MCI compared to healthy older adults. However, meta-analysis findings suggest that older adults with MCI had statistically non-significant yet lower differences in QOL compared to their healthy counterparts.

**Conclusion:**

Future research should focus on developing QOL assessment tools specifically for older adults with MCI and follow-up studies that could provide better knowledge of their changing cognitive profile and life quality.

## RESUMO

O aumento global da população idosa tem suscitado preocupações quanto a condições relacionadas à idade, como demência e comprometimento cognitivo leve (CCL), e seus impactos nas pessoas afetadas. Indivíduos com CCL apresentam déficits cognitivos superiores ao esperado para sua idade e nível de alfabetização, e embora a condição seja considerada leve estudos demonstram que até déficits mais sutis podem afetar a qualidade de vida (QdV).

**Objetivo:**

Este trabalho explora e compara a QdV em idosos com e sem CCL, por meio de revisão sistemática e metanálise.

**Métodos:**

Após uma busca detalhada de artigos até maio de 2021 em relevantes bases eletrônicas (PubMed Central, PubMed, Scopus, CINAHL Plus, Web of Science, ProQuest e Cochrane) usando as palavras-chave *"mild cognitive impairment", "quality of life", "old", "old aged", "aged", "older adult", "geriatrics", "healthy controls", "healthy participants",* e "*normal controls*", incluímos 23 artigos na revisão e 12 na metanálise.

**Resultados:**

A qualidade dos artigos foi avaliada com a ferramenta Modified Downs and Black. A revisão revelou diferenças nas pontuações de QdV entre idosos com CCL e saudáveis, mas a metanálise revelou diferenças estatisticamente não significativas, sugerindo uma QdV ligeiramente inferior nos primeiros.

**Conclusão:**

Futuras pesquisas devem enfocar ferramentas de avaliação de QdV específicas para idosos com CCL e em estudos de acompanhamento para melhor compreensão do perfil cognitivo em evolução e da qualidade de vida desses indivíduos.

## INTRODUCTION

Aging is a biological, irreversible, and inevitable phenomenon. Recent advances in medical and healthcare services have considerably increased human longevity. Globally, the average life expectancy has increased from 66.8 years in 2000 to 73.4 years in 2019, an increase of almost six years over the past two decades^1^.

Aging is generally associated with a decline in various cognitive functions such as memory, attention, executive functions, and processing speed, to name a few. Clinically, there exists a progressive decline from normal aging to cognitive decline, such as in Alzheimer's dementia type^2^. The boundary between normal aging and (early) Alzheimer's disease (AD) is widely researched. It remains an active area of inquiry due to the daily difficulties these older adult populations face. Some individuals exhibit a transitional state between normal aging and dementia, known as mild cognitive impairment (MCI), where cognitive skills are preserved enough to perform daily activities independently, yet with frequent interruptions.

The term "mild cognitive decline" was initially used in the third stage of a global deterioration scale while explaining the clinical characteristics of primary degenerative dementia progression^3^. The term MCI gained popularity after Petersen et al.^4^ revived it. According to Petersen et al.^4^, the diagnostic criteria for MCI are:

- memory complaint, preferably corroborated by an informant;
- memory impairment relative to age- and education-matched healthy people;
- preserved general cognitive function;
- preserved activities of daily living; and
- not clinically demented. More recently, the KeY Symposium 2004 criteria proposed the classification of MCI as amnestic and non-amnestic based on memory impairment, and further distinguished these types of MCI as single domain or multiple domains depending on the cognitive domains affected. MCI is a syndrome defined as a cognitive decline greater than expected for an individual's age and education level but that does not interfere *profusely* with daily life activities^5^.

Earlier reports on the prevalence of MCI among older adults aged ≥65 years ranged from 3.0 to 17.0%^6^. A meta-analysis of epidemiological studies and a systematic review of the Chinese older adult population aged ≥60 years showed a pooled prevalence of 12.7% and 14.7%, respectively^7,8^. More recent studies indicated a higher prevalence of MCI in the older population as in the case of a Swedish article that estimated a prevalence ranging from 5.1 to 29.9% in individuals aged ≥60 years^9^. Similarly, a study from Saudi Arabia reported a remarkably high (38.6%) prevalence of MCI in older adults^10^.

Clinical monitoring of individuals with MCI is crucial due to the high risk of progressing to dementia. The annual conversion rate of MCI to dementia is approximately 10–15%, in contrast to 1–2% in the healthy older population below 80 years of age^11^. That is, people with MCI are 2.8 times more likely to progress to AD than their cognitively healthy counterparts^12^.

Cognitive deficits, irrespective of their severity, are associated with a decline in quality of life (QOL)^13^. The World Health Organization (WHOQOL Group, 1995) defines QOL as "an individual's perception of life in the context of culture and value system in which he or she lives and in relation to his or her goals, expectations, standards, and concerns". It is regarded as a good indicator of an individual's overall health and reflects their physical, mental, and social health and their impact on life. Research studies emphasize the assessment of QOL in persons with cognitive deficit^14,15^ as even those with MCI^13^ or self-perceived cognitive impairment^16^ have been reported to experience reduced QOL compared to the healthy older adult population.

Given the change in awareness of cognitive functions from normal cognition to dementia stage, it is fundamental to study the QOL in MCI which forms the initial stages of cognitive decline. Awareness of cognitive decline (ACD) studies has led to conflicting results where both individuals with low ACD and memory high complaints had an increased risk of having positive AD biomarkers. Individuals’ awareness of their cognitive changes experienced at the initial stages is high, but this awareness decreases soon, with them exhibiting mild anosognosia in the MCI stage and severe anosognosia in dementia^17^.

Many studies have explored QOL in normal controls and individuals with MCI, dementia, and AD. However, when they studied the relationship between cognitive decline and QOL across the continuum from normal to dementia, it was observed that there exists a robust relationship between impairments in QOL and cognitive complaints. This warrants future studies that will help in early diagnosis and management of these clinical populations^18^.

Considering that the older adult population is on the rise with the substantial increase in the number of people with MCI, there is a need to understand whether the QOL deteriorates in persons with MCI compared to healthy controls. For this purpose, we performed a systematic review and meta-analysis of the QOL in the elderly with and without MCI.

## METHODS

### Search process

The current systematic review and meta-analysis were performed according to the Preferred Reporting Items for Systematic Reviews and Meta-Analyses (PRISMA) guidelines. This study is registered on the International Prospective Register of Systematic Reviews (PROSPERO), under CRD42018109302. We reviewed studies on the QOL in older adults with MCI. A detailed search of relevant articles was carried out in the following electronic databases: United States National Library of Medicine (PubMed) Central, PubMed, Scopus, Cumulative Index to Nursing and Allied Health (CINAHL) Plus, Web of Science, ProQuest, and Cochrane in May 2021. The keywords used for the search were "mild cognitive impairment", "quality of life", "old", "old aged", "aged", "older adult", "geriatrics", "healthy controls", "healthy participants", and "normal controls". All these keywords were used in different combinations along with the Boolean operators "AND" and "OR" to identify pertinent studies. We did not set a restriction on the year of publication; thus, all articles published up to the end of the search period were considered for review and analysis. Additionally, the reference list of all selected articles was screened for any eligible articles that could be included in the study.

### Selection of studies

For the systematic review, we included those studies where:

- the mean age of the participants was ≥50 years;
- the diagnosis of MCI was established with a proper tool;
- healthy participants were included as a control group; and
- the QOL was considered as an outcome measure.

In this review, QOL refers to an individual's overall health which is determined by various domains such as physical, mental, and social health. An older adult with impairments in any of the cognitive domains (e.g., executive function, attention, language, orientation) including memory, despite preserved activities of daily living and absence of dementia, as assessed using any objective screening tool or available diagnostic criteria, is considered to have MCI. All eligible cross-sectional and cohort studies were included in this review. Further, we excluded all articles that reported information collected exclusively from caregivers, those published in a language other than English, and all reviews. Among the studies selected for the systematic review, those with quantitative data were considered for the meta-analysis. All the articles obtained through the search were uploaded to Rayyan QCRI^19^. The first two authors were involved in the selection of studies by screening titles and abstracts, and any discrepancies in the inclusion were sorted by the last author.

### Data extraction

The first author extracted the authors’ details, publication year, sample size, age of the participants, instruments used for QOL assessment (outcome measure), mean and standard deviation (SD) scores, and study results. The extracted data were entered into a spreadsheet (Microsoft Excel) for later review and analysis.

### Quality assessment

This study's three authors (1, 2, and 4) were involved in the quality assessment. The first two independently assessed the quality of all selected articles with the Modified Downs and Black tool^20^. This checklist tool consists of 28 questions and assesses the quality of the studies in reporting, external validity, bias, confounding, and power. The score of each question was either 1 ("yes") or 0 ("no" or "unable to determine"). Among the 28 questions, 14 did not apply to the studies reviewed here (Supplementary Material – https://www.demneuropsy.com.br/wp-content/uploads/2024/02/DN-2023-0093-Supplementary-Material.docx). The eliminated questions were applied to randomized controlled studies but not to cross-sectional studies. Hence, the maximum possible score was 14. The discrepancies in the rating of the checklist by the first two authors were sorted out by the fourth author. We employed an ad-hoc criterion for categorizing the articles based on their quality scores: 1–4=poor, 5–9=fair, and 10–14=good.

## RESULTS

### Description of studies

The search process (see Methods section) returned a total of 6,791 articles. We identified and removed 171 duplicates from the complete set. Title screening was performed for further scrutiny, and 95 articles were selected. After reviewing the abstracts of these selected articles based on the eligibility criteria, 73 were subjected to full-text screening. Of these, 50 articles were excluded for presenting review studies, due to different clinical populations, different outcome measures, etc. (Supplementary Material 2 – https://www.demneuropsy.com.br/wp-content/uploads/2024/02/DN-2023-0093-Supplementary-Material-2.docx). Thus, 23 full-text articles that met the eligibility criteria were included in the present systematic review (Table 1)^13,18,21–41^.

**Table 1:** Main characteristics of the studies included in this systematic review and meta-analysis.

Reference	Year	Age (years)	Sample size	Study design	Tools for assessing MCI	Tools for assessing QOL	Outcome measures
Bárrios et al.^13^	2013	HC 68.1±9.5 MCI 71.7 ± 8.1	HC=22 MCI=30	Translation and validation in Portugal	MMSE	QOL-AD- (Portuguese and European Portuguese versions	QOL
Bárrios et al.^34^	2013	HC 66.3±10.8 MCI 70.8±6.2	HC=26 MCI=25	Cross sectional	MMSE	QOL-AD (Portuguese)	QOL
Calero and Navarro^28^	2011	65–96	HC=141 MCI=79	Cross sectional	MMSE (Spanish)	CUBRECAVI	QOL
Chang et al.^35^	2017	≥60 80.9±6.1	HC=251 MCI=48	Correlational One to one interview	SPMSQ	WHOQOL–BREF- Taiwanese version	Prevalence and distribution of MCI Correlation between MCI and QOL
Hussenoeder et al.^36^	2020	N=86.3±2.9 MCI 87.9±3.8	HC=793 MCI=110	Prospective longitudinal study	Criteria proposed by International Working Group on MCI	WHOQOL-OLD	QOL
Johansson et al.^23^	2012	85	HC=265 MCI=91	Part of a population study ELSA 85	MMSE	EQ-5D	HRQOL
Kameyama et al.^24^	2016	HC 82.1±5.3 MCI 85.2±4.3	HC=11 MCI=14	Cross sectional	MMSE	EQ-5D	HRQOL
Karademas et al.^30^	2019	HC 66.6±7.44 MCI 72.7±8.2	HC=225 MCI=127	Cross sectional	Modified Petersen criteria	WHOQOL-BREF	QOL
Lapid et al.^22^	2011	90–99 HC 93.3±2.5 MCI 93.9±2.8	HC=56 MCI=13	Prospective	MMSE STMS	LASA	QOL
Lin et al.^31^	2017	≥50 years	HC=100 aMCI=125 naMCI=61	Cross sectional	MMSE	QOL-AD	QOL
Maki et al.^29^	2014	≥ 65 HC 71.9±4.1 MCI 73.1±4.4	HC=120 MCI=37	Cross sectional	MCI was diagnosed by a physician. MMSE 5-Cog test: memory test and executive function test	SDL Japanese version	Impacts of memory complaints on QOL
Missotten et al.^27^	2008	≥ 65 HC 79.2±6.76 MCI 83.7±7.0	HC=72 MCI=36	Cross sectional	MMSE Petersen criteria	ADRQL	Sensitivity of ADRQL
Muangpaisan et al.^32^	2008	≥ 50 HC 63.9±7.9 MCI 66.7±8.0	HC=37 MCI=85	Cross sectional	Petersen criteria	WHOQOL-BREF-Thai version	QOL
Onandia-Hinchado and Diaz-Orueta^41^	2019	HC 75.0±6.7 (62–91 years) MCI 70.8±11.5 (54–85 years)	HC=31 MCI=43	Cross sectional	Criteria from SEN	SF12v2	HRQOL
Onandia-Hinchado and Diaz-Orueta^37^	2019	HC 74.7±6.35 MCI 70.9±11.1	HC=31 MCI=37	Cross sectional	MMSE	SF12v2	HRQOL
Parsaik et al.^21^	2012	90–99 HC 92.0 (92.0, 95.0) MCI 93.0 (93.0, 96.0)	HC=45 MCI=13	Cross sectional cohort	STMS, MMSE, DRS	LASA	QOL
Pusswald et al.^38^	2015	HC 66.9± 9.4 naMCI 67.1±9.6 aMCI 68.9±9.0	HC=98 naMCI=98 aMCI=98	Cross Sectional	Petersen Criteria MMSE MoCA	SF-36	HRQOL
Pusswald et al.^39^	2016	HC 66.0 (60–72) naMCI 66.0 (59–72) aMCI 68 (60–74)	HC=317 naMCI=297 aMCI=224	Cross Sectional	Petersen Criteria MMSE MoCA	SF-36	HRQOL
Pusswald et al.^40^	2019	HC 64.4±5.5 MCI 65.2±6.8	HC=20 MCI=37	Part of a prospective cohort study	MMSE	SF-36	HRQOL
Ready et al.^26^	2004	60–91 HC 74.7±6.8 MCI 77.4±6.9	HC=23 MCI=30	Cross sectional	MMSE Petersen criteria	DQOL	QOL
Stites et al.^25^	2017	≥ 65 HC 79.2±7.1 MCI 78.1±6.4	HC = 99 MCI=92	Recruited from longitudinal cohort study	NACC and Petersen criteria MMSE	EQ-5D EQ-VAS QOL-AD DEMQOL	QOL
Stites et al.^18^	2018	≥ 65 HC 79.2±7.1 MCI 78.1±6.4	HC= 99 MCI=92	Recruited from longitudinal cohort study	NACC and Petersen criteria MMSE	EQ-5D EQ-VAS QOL-AD DEMQOL	QOL
Teng et al.^33^	2012	HC 70.1±8.6 MCI 72.0±9.5	HC=97 MCI=108	Cross sectional	MMSE	QOL-AD	QOL

MCI: mild cognitive impairment; QOL: quality of life; HC: healthy controls; MMSE: Mini-Mental State Examination; QOL-AD: Quality of Life Alzheimer's Disease; CUBRECAVI: Short QoL questionnaire; SPMSQ: Short Portable Mental Status Questionnaire; WHOQOL-BREF: World Health Organization Quality of Life brief version; WHOQOL-OLD: developed for older adults; DQOL: Dementia Quality of Life Questionnaire; NAAC: National Alzheimer's Coordinating Center; EQ-5D: Euro Quality of Life of five dimensions; ELSA: Longitudinal Adult Health Study; HRQOL: Health-related Quality of Life; STMS: Short Test of Mental Status; LASA: Linear Analogue Self-Assessment Scale; SDL: Satisfaction in Daily Life; ADRQL: Alzheimer's Disease Related Quality of Life; SEN: Spanish Society of Neurology; SF12v2: 12-Item Short Form Health Survey, version 2; DRS: Dementia Rating Scale; MoCA: Montreal Cognitive Assessment; SF-36: Short Form-36 Questionnaire; EQ-VAS: Euro Quality of Life -Visual Analogue Scale; DEMQOL: Dementia-Related Quality of Life;

Of these 23 studies, 19 were cross-sectional and four were longitudinal cohort studies. Based on the scores obtained from the Modified Downs and Black checklist (Supplementary Material 2 – https://www.demneuropsy.com.br/wp-content/uploads/2024/02/DN-2023-0093-Supplementary-Material-2.docx), the quality of 18 articles was good (10–14), and that of five articles was fair (5–9).

### Study participants

The 23 selected studies included 2,050 older adults with MCI and 2,979 healthy controls (HC) aged 50–99 years. Two studies^21,22^ recruited participants between 90 and 99 years old. Another study^23^ included participants aged 85 years (only) as it was part of a population study. Regarding gender, most studies included participants from both gender groups, except for one^24^ that recruited only older women based on its objectives. Most of the participants from the studies included in this review were diagnosed based on the criteria proposed by Petersen et al.^11^, the Spanish Society of Neurology (SEN), and the National Alzheimer's Coordinating Center (NACC), and a few were based on the scores obtained from cognitive assessment tools such as Mini-Mental State Examination (MMSE)^42^, Dementia Rating Scale (DRS)^43^, Short Test of Mental Status (STMS)^44^, Montreal Cognitive Assessment (MoCA)^45^, Short Portable Mental Status Questionnaire (SPMSQ)^46^, and 5-Cog test^47^. In most of the studies, participants with MCI diagnosis were recruited. In a few other studies, older adults were screened for MCI using standardized tools as mentioned above and recruited after fulfilling the criteria.

### Outcome measures

The reviewed studies employed various tools to measure QOL in older adults with and without MCI. Twenty-one studies reported QOL based on a single instrument, whereas two studies used more than one^18,25^. The QOL instruments used in the studies were the Linear Analogue Self-Assessment Scale (LASA)^48^, Short Form-36 Questionnaire (SF-36) – Iranian version^49^, WHOQOL-BREF (the brief version) — Greek^50^ and WHOQOL-BREF – Taiwanese^51^ and Thai^52^ versions, Dementia Quality of Life questionnaire (DQoL)^53^, Quality of Life Alzheimer's disease (QOL-AD) – Portuguese version^54^, QOL-AD^55^, Alzheimer's Disease Related Quality of Life (ADRQL)^56^, the Japanese version of Satisfaction in Daily Life (SDL)^57^, Dementia-Related Quality of Life (DEMQOL)^58^, Euro QOL of five dimensions (EQ-5D) and of visual analogue scale (EQ-VAS)^59^, CUBRECAVI – *Cuestionario breve de calidad de vida* (Short QoL questionnaire)^60^, WHOQOL-OLD (developed for older adults)^61^, 12-Item Short Form Health Survey, version 2 (SF12v2) questionnaire^62^ (Table 2).

**Table 2:** Profile of tools including domains used for assessment of quality of life in persons with mild cognitive impairment.

QOL Measure	Domains	Score
SF-36	physical functioning, role limitations due to physical health, pain, general health, energy/fatigue, social functioning, role limitations due to emotional problems, emotional well-being	0–100
EQ-5D	mobility, self-care, usual activities, pain/discomfort, anxiety/depression	Index value +1 to −1
WHOQOL-BREF	physical health, psychological state, social relationships, environmental context	5-point Likert scale
DQOL	positive affect, negative affect, feelings of belonging, self-esteem, sense of aesthetics	5-point Likert scale
QOL-AD	physical health, energy, mood, living situation, memory, family, marriage or close relationship, friends, self as a whole, ability to do chores around the house, ability to do things for fun, money, life as a whole	4-point Likert scale
ADRQL	social Interaction, awareness of self, feelings and mood, enjoyment of activities, response to surroundings	0–100%
SDL	physical health, mental health, self-care, gait, housework, house facilities, partner and family relationship, hobby and leisure activities, social interaction, economic state and social security, job satisfaction	5-point Likert scale
LASA	physical well-being, emotional state, faith, religious involvement, intellectual state, social interactions, pain frequency, pain intensity, coping ability	10-point Likert scale
HRQOL	Single-item overall quality-of-life rating	5-point Likert scale
WHOQOL- OLD	sensory abilities; autonomy; past, present, and future activities; social participation; fears related to death and dying; and intimacy	0–100
DEMQOL	Degree of difficulty in daily life related to health, well-being, cognitive functioning, social relationships, daily activities, and self-concept	0–100
CUBRECAVI	health (subjective, objective and psychic); social integration; functional abilities; activity and leisure time; quality of environment; satisfaction with life; education; income; health and social services	5-point Likert scale
SF12	Physical Function, Physical Role, Body Pain, General Health, Vitality, Social Function, Emotional Role, and Mental Health	0–100

### Meta-analysis

For the meta-analysis, we selected 12 of the 23 articles included in the systematic review. Eleven articles were excluded from the meta-analysis due to the lack of primary data (mean and SD). As two studies^18,25^ used multiple tools to assess the QOL in older adults, each tool's outcome was considered a separate study for the meta-analysis. The meta-analysis was undertaken to pool the mean QOL scores provided in each study to obtain standardized mean differences for both groups. A random effects model with an inverse variance weighting scheme that provides weightage inversely proportional to the standard error of the estimate was applied to the analysis. The standard error of the mean (SEM) was computed as, where SD denotes the standard deviation and ‘n’ indicates sample size. Chi-squared or Q statistic was used to determine the statistical significance of heterogeneity, and I-squared statistic was computed to express heterogeneity as a percentage. The meta-analysis was performed in the STATA v. 13.1 statistical software using the Multi-Environment Trial Analysis (METAN) package^63^ and set the level of significance at 5%.

**Figure 1:**
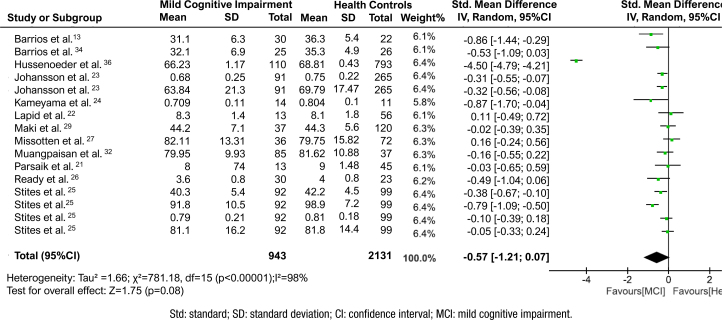
Forest plot of mean QOL scores compiled from all the studies included in meta-analysis. Stites et al.^25^ refers to the outcome measures data from the same study with different tools.

Figure 1 (Forest plot) shows the results of the meta-analysis. It provides the author's name, year of publication, sample size, and standard mean differences with a 95% confidence interval, (CI) and the percentage weightage received for each study. Overall mean QOL with 95%CI is depicted as a diamond. Chi-squared and I-squared statistics are also depicted in the plot. The overall QOL did not show any statistically significant changes compared to the HC (SMD −0.57; 95%CI −1.21 to 0.07; participants=943+2,131; studies=16; I^2^=98%). The level of heterogeneity was I^2^=98%; p<0.00001 (Figure 1).

The funnel plot was applied, which indicated the presence of publication bias.

## DISCUSSION

The current work aimed to compare QOL in older adults with and without MCI through a systematic review (HC=2,979, MCI=2,050) and a meta-analysis (HC=2,131, MCI=943). Most of the studies included were cross-sectional except for four longitudinal cohort studies. All of them compared the QOL of older adults with and without MCI using a diverse set of tools. Among the 23 studies included in this systematic review, 15 reported significantly low QOL scores in older adults with MCI compared to HC. In contrast, the remaining eight studies reported no differences between the groups^21,22,26–31^.

Based on the meta-analysis, the QOL in older adults with MCI showed lower scores than in HC. However, the difference was not statistically significant. The literature review showed inconsistent findings on systematic review and meta-analysis data of the QOL in healthy and MCI older adults. Studies on health-related QOL in an Iranian population revealed that the mean QOL scores in healthy older adults were 53.8^64^ and 54.9^65^. Both studies used the SF-36 questionnaire for the health-related QOL assessment, and the scores were low compared to those obtained from other countries, especially economically developed nations.

The QOL in older adults with MCI remained inconclusive, with a few studies reporting no differences in cognitive functioning between the MCI group and HC. However, from the results of this study, it may be noted that seven of the 23 selected studies reported no^21,22,26,27,29,32^ or weak^23^ correlation between cognitive functions and QOL. Several reasons have been attributed to the differences in the results, including assessment tools used, sources of data collection, and assessment methods. One of the reasons for the lack of (or weak) correlation between the QOL and cognitive functions was the use of instruments that were not sufficiently sensitive to detect MCI in these participants (e.g., MMSE and Saint Louis University Mental Status [SLUMS] Examination). The MMSE, a screening instrument, is not adequately sensitive to rule out MCI^23^. Moreover, the assessment of the QOL through postal questionnaires has also been reportedly ineffective in persons with MCI^23^. The lack of clear cut-off values in certain studies on the QOL in persons with MCI^23^ leaves the outcomes difficult to interpret. A study^22^ that reported no correlation between QOL and cognitive functions recruited participants in the age range of 90–99 years. These authors argued that the reason for good QOL even at an older age was due to their participants’ (centenarians’) experiences in early life that enabled them to tackle adverse situations in later life and lead a good QOL.

A few studies that collected data from the community setup^26^ and memory clinic^32^ did not find any correlation between QOL and cognitive impairment in MCI older adults. Further, participants with better insight into their cognitive status rated their QOL as poorer than those unaware of their cognitive condition^25,29^. Comparisons between self-rating and ratings by informants/caregivers showed a lack of agreement between them^26^. The ratings by the caregivers were poorer than the self-ratings. This finding signifies the role of the participants’ insight in the QOL ratings. Though we included data from the informants in this review and analysis, our results corroborate earlier studies that state that older adults with MCI experience poor QOL^13,33^.

In the domain of the assessment of QOL, both generic and disease-specific tools were in use. While the generic tools were used to assess the QOL in both healthy and clinical populations, the disease-specific tools were intended to measure the QOL in specific clinical populations such as individuals with dementia. Among the available generic tools, the SF-36 health survey has been the most commonly used QOL assessment tool in individuals with MCI. However, based on the search of literature, a wide range of instruments were used for QOL assessment in older adults with MCI^66^. The selection of one or more tools depends on the purpose of the assessment and factors such as the characteristics of the population and the environment where the measurements are carried out^67^. Along with the SF-36 tool, which is recommended for a detailed assessment of QOL, the QOL-EQ-5D was also reported to have good validity and reliability in evaluation^68^. The studies included in this review used a range of tools to assess QOL, and the most employed generic tools were LASA, SF-36, WHOQOL, WHOQOL-BREF, SDL, CUBRECAVI, SF12v2, and WHOQOL-OLD. The most popularly used disease-specific tools included DEMQOL, QOL-AD, DQL, and ADRQL for measuring QOL in MCI individuals. Among these specific tools, the QOL-AD has been reported to be a valid and reliable tool in patients with cognitive impairment whose MMSE scores ranged from 10–29^69^. Only a small proportion of studies employed life satisfaction tools (e.g., SDL^57^) for assessing QOL in MCI persons. However, none of these tools were explicitly developed for the older population. Most of them are more suitable for younger adults. Such tools may be insensitive to more critical areas for older adults. Another concern is the scarcity of specific tools to assess QOL in individuals above 60 years of age. In this review, Hussenoeder et al.^36^ used one such tool (WHOQOL-OLD^61^) to assess QOL in the elderly population. In this context, caution must be exercised while interpreting the outcomes of those studies that used tools that were not designed to measure QOL in the older adult population^70^.

Besides the factors mentioned above, the domains covered in these QOL assessment tools were quite different, as each was developed to probe into domains like overall health or domains related to specific diseases like Alzheimer's (Table 2). Even among the generic tools, the domains covered were not uniform. The WHOQOL-BREF and SDL include questions that probe into the domains of physical health, psychological health, social well-being, and the environmental context. However, the EQ-5D and SF-36 do not cover most of those domains, restricting their questions to mobility, self-care, and pain. Among the disease-specific tools, QOL-AD covers all the domains that influence QOL, which could be attributed to the disease-specific nature of this tool. However, the other two disease-specific tools, DQOL and ADRQL, address particular domains such as the person's mental status and performance abilities, etc. LASA^25^ is the only tool that probes into the details of pain and coping ability that is not covered by other tools. WHOQOL-OLD was developed specifically to address older adults’ questions related to autonomy and death, which are not addressed by other tools.

The scoring was subjective and varied across the QOL tools, which could have possibly influenced the overall QOL ratings. Each item in the tool was rated with Likert scales (5-point: WHOQOL-BREF, WHOQOL-OLD, DQOL, SDL, and CUBRECAVI; 10-point: LASA; and 4-point: QOL-AD, DEMQOL). The SF-36, SF12v2, and EQ-VAS, on the other hand, provided scores in the range from 0 (worst imaginable health state) to 100 (best imaginable health state). The scoring of ADRQL ranged from 0 to 100%, and that of the EQ-5D was converted into a single index score ranging from +1 to −1, where +1 represents perfect health, 0 indicates a state equivalent to death, and −1 indicates worse than death.

Since persons with MCI may not present obvious complaints such as pain and inability to perform their daily activities, disease-specific tools may not be appropriate to measure QOL in this population. Notably, this review evidenced the heterogeneity in the instruments used to assess the QOL in older adults with MCI. The domains included in these tools were different, making the generalization of findings and sub-domain analysis difficult. Given the heterogeneity in the tools used to assess QOL in MCI literature, future reviews may consider assessing QOL in MCI using a single or most commonly used tool, or comparing QOL in MCI using existing tools. Since there are no standard protocols followed for the assessment of QOL, this limitation is inevitable. Thus, this review clearly demonstrated the need for a specific tool for assessing QOL in MCI, specifically in older adults, and a need for future research in this field. Having appropriate tools will help us enhance the intervention programs currently available, thereby improving the QOL in older adults with MCI.

In conclusion, this systematic review and meta-analysis revealed statistically non-significant results wherein older adults with a mild decline in cognitive functions did not have negative effects on the QOL when compared to those without it. Being a vital domain, periodic assessment of the QOL is essential for older adults with MCI to monitor their cognitive status. This will help us plan meaningful intervention programs that require specific tools designed for this purpose, a potential need of the hour in the population with MCI.

### Limitations

This review study has some limitations that need to be considered while interpreting the results. Some of them are limited databases used for search, including studies in the English language only and not assessing gray literature. Further, our review did not include subgroup analysis based on types of MCI, tools used for assessment, or age groups, which could have expanded our knowledge. Subgroup analysis could not be carried out due to the inadequate number of studies. Another limitation of this review is the lack of meta-regression with age being considered as a regression variable. Since there is an indication of publication bias, the results should be considered cautiously.
